# Improving Emergency Medical Services (EMS) Care for People With Autism in the Prehospital Setting Through Sensory and Communication Aids

**DOI:** 10.7759/cureus.74702

**Published:** 2024-11-28

**Authors:** Eric Lombardi, Fred Lepore, Collin Greer

**Affiliations:** 1 Emergency Medicine, Franciscan Crown Point Health, Crown Point, USA; 2 Emergency Medicine, Franciscan Health Dyer, Dyer, USA; 3 Emergency Medicine, Franciscan Health Olympia Fields, Olympia Fields, USA

**Keywords:** autism spectrum disorder (asd), emergency medicine, emergency medicine barriers, ems training, prehospital ems, research in emergency medicine, sensory disorder

## Abstract

Autism spectrum disorder (ASD) is a prevalent neurodevelopmental disorder characterized by social, communication, and behavioral challenges. Emergency medical services (EMS) environments, with their loud noises, bright lights, and unfamiliar personnel, often exacerbate these challenges, making care for individuals with ASD particularly complex. To address these challenges, the Franciscan Crown Point EMS system introduced the “Ben’s Blue Bags” (BBBs) program. These bags, available on every transport vehicle, contain sensory aids, headphones, a dry erase board for communication with nonverbal patients, and a pictogram of the human body for identifying injuries. The BBB program serves 18 different municipalities across four counties in Northwest Indiana, covering over 200 paramedics, 500 emergency medical technicians (EMTs), and 60 transport vehicles in both rural and urban settings.

Over an eight-month period in 2023, 77 EMS providers participated in a survey assessing the effectiveness of BBBs. The results indicate that 94.2% of respondents found BBBs enhanced their ability to deliver high-quality care to individuals with ASD. Sensory aids were deemed the most valuable component, helping calm patients and reduce anxiety during EMS interventions. On a scale from 1 to 10, the mean rating was 9.08, with a standard deviation of 1.757, signifying that BBBs were "overwhelmingly helpful." All but three respondents reported that BBBs were helpful in keeping individuals with ASD calm during interactions, and 58 out of 65 paramedics found BBBs useful for calming and/or distracting patients to enable intravenous (IV) placement. Additionally, 61 respondents reported at least one instance where an unnecessary emergency room visit was avoided due to help from BBBs.

This study concludes that sensory aids and communication tools can significantly improve prehospital care for patients with ASD. We recommend the broader implementation of such tools and further research to explore their impact within EMS and emergency medicine.

## Introduction

Autism spectrum disorder (ASD) is a lifelong neurodevelopmental condition affecting approximately one in 59 children [1,2]. Characterized by difficulties in social interaction, communication, and behavior, individuals with ASD often display restricted and repetitive behaviors, labile emotions, and episodes of acute agitation [2]. The exact etiology of ASD remains largely unknown, though it is believed to result from a complex interplay of genetic and environmental factors [3]. The wide variability in symptoms among individuals with ASD poses challenges in developing universal care solutions [4].

Emergency medical services (EMS) face particular difficulties when providing care to individuals with ASD. The EMS environment - characterized by unfamiliar people, loud noises, bright lights, confined spaces, and the need for medical interventions - can induce significant distress, exacerbating symptoms such as stimming, heightened anxiety, and mental breakdowns [5,6].

To address these challenges, our EMS system in Northwest Indiana implemented the “Ben’s Blue Bags” (BBB) program. Each bag contains sensory aids including headphones to reduce ambient noise, a dry erase board for communication with nonverbal patients, a pictogram for indicating pain locations, and various sensory items such as stress squeeze balls, Rubik's cubes, bendable bracelets, “pop-its,” and fidget spinners. The program was deployed across all ambulances in our service area to improve prehospital care for patients with ASD.

Study objectives

This study aims to evaluate the efficacy of multisensory aids in enhancing care for individuals with ASD in the prehospital setting. The theoretical foundation of this study posits that the unique care requirements of autistic patients can be better addressed using sensory and communication aids. Many individuals with ASD may have distinct care needs compared to the general population, such as being nonverbal or experiencing extreme difficulty when exposed to new stimuli or deviations from routine activities.

The primary goal of this study is to assess the effectiveness of sensory aid tools in addressing these unique care requirements from both the patient’s and EMS provider’s perspectives. Additionally, this study seeks to provide data supporting the use of such tools and to raise awareness of the need for further research into their role in prehospital care. Ultimately, the study aims to encourage the adoption of these techniques in EMS departments across the nation.

## Materials and methods

"Ben's Blue Bags" (BBB) are blue vinyl bags, available on every transport vehicle in the system, which contain sensory aids including noise-reducing headphones, dry erase boards for nonverbal communication, human body pictogram to illustrate where the patient is injured, and sensory items such as stress balls and fidget spinners. Each transport service purchased a BBB for the total cost of $200 per bag. The individual sensory aids in the bags were purchased from online vendors and are one-time-use disposable aids.

A voluntary, anonymous survey was conducted to evaluate the impact of the BBB program on prehospital care for individuals with autism spectrum disorder (ASD). This study was performed by EMS providers within the Franciscan Crown Point EMS system wherein BBB have been utilized. The Franciscan Crown Point EMS system spans 18 service lines across Lake, Newton, and Jasper counties in Northwest Indiana. The system includes both urban and rural areas, covering over 200 advanced life support (ALS) paramedics, 500 basic life support (BLS) emergency medicine technicians (EMTs), and more than 60 transport vehicles. 

The survey consisted of both quantitative and qualitative items designed to assess the perceived effectiveness of the BBB kits. Participants were asked to rate the kits on a 10-point Likert scale, with scores ranging from 1 (“helped a little”) to 10 (“incredibly helpful”). They were also asked to indicate which components of the kits were most useful. 

Surveys were distributed online from February to September 2023. Respondents included paramedics, EMTs, firefighters, and police officers within the EMS system. Participation was voluntary, and no personally identifiable information was collected.

## Results

**Figure 1:**
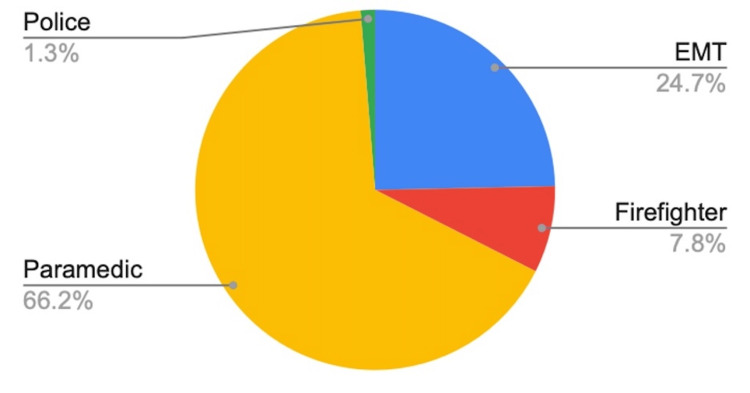
First Responders by Category

A total of 77 personnel participated in the survey, representing a diverse range of prehospital care providers, including paramedics (n=51), EMTs (n=19), firefighters (n=6), and one police officer (see Figure 1).

Effectiveness of Ben's Blue Bag kits 

**Figure 2:**
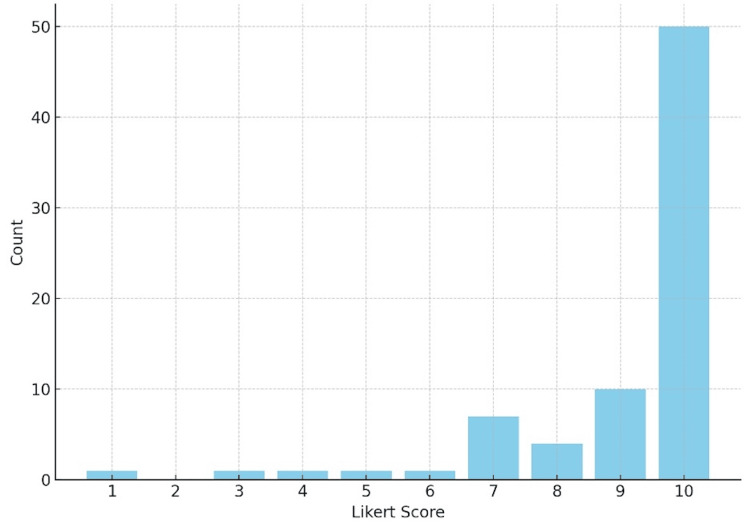
BBB Overall Helpfulness Likert Score BBB: Ben's Blue Bags

**Figure 3:**
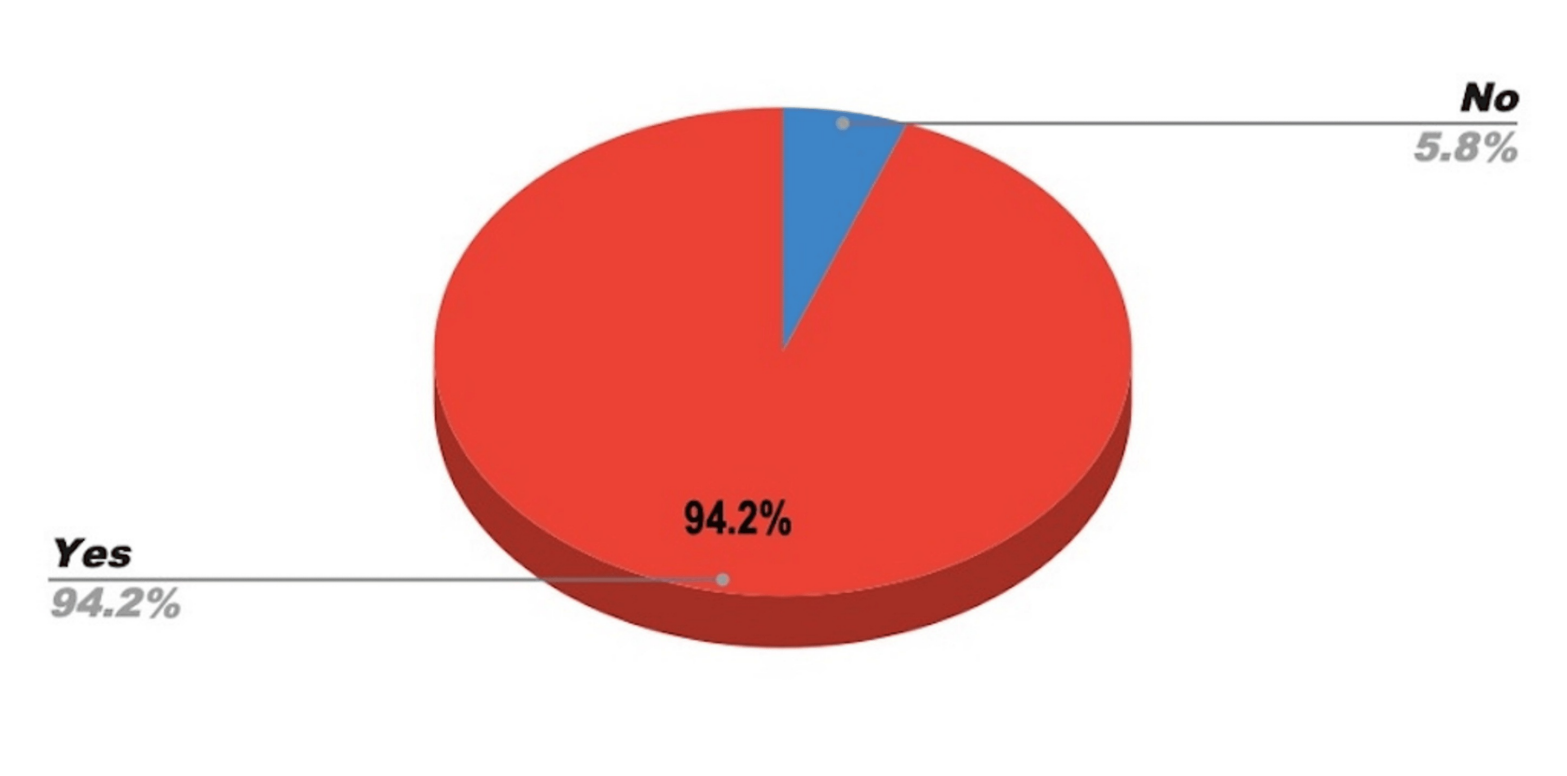
Did BBB Help Provider Give a High Level of Medical Care to Autistic Persons? BBB: Ben's Blue Bags

On a 10-point scale, participants rated the overall effectiveness of the BBB kits with a mean score of 9.08 (standard deviation = 1.757), indicating high levels of perceived utility. Among respondents, 94.2% reported that the kits improved their ability to deliver high-quality care to individuals with ASD. Only four respondents indicated the kits did not significantly enhance their practice (see Figures 2,3).

Specific use cases 

**Figure 4:**
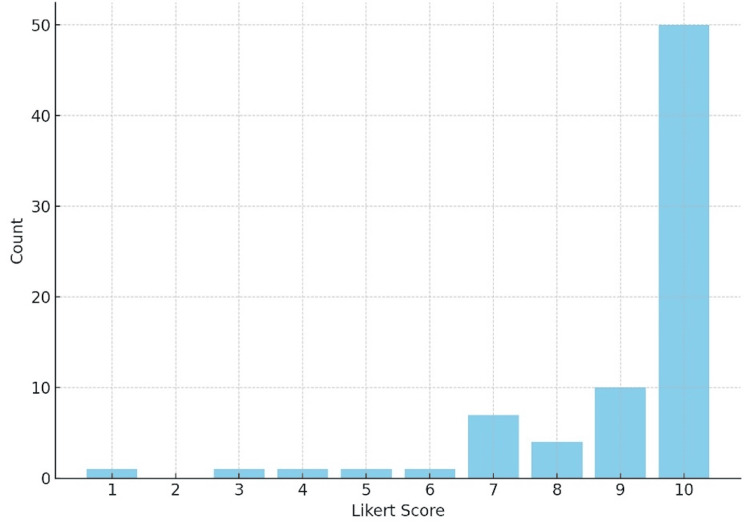
IV (Intravenous) Placement Helpfulness Likert Score

*Patient Calming and Communication*: All but three respondents found the BBB kits helpful in keeping individuals with ASD calm during EMS interactions. Specifically, 58 out of 65 paramedics reported that the kits were effective in calming or distracting patients during intravenous (IV) placements (see Figure 4).

*Avoidance of Unnecessary ER Visits*: Notably, 61 respondents (77.9%) reported using the kits to de-escalate ASD-related meltdowns, which helped avoid at least one unnecessary emergency department visit. Of these, 21% avoided two or more unnecessary visits.

Most useful kit components 

Participants identified sensory aids as the most valuable part of the BBB kits. Sensory items such as stress balls and fidget spinners were identified as most valuable by 63.6% (n=49) of EMS providers surveyed. Followed by noise-reducing headphones chosen by 26% (n=20) as the most valuable component of BBB kit. The third most valuate was dry erase boards, which were preferred by 6.5% (n=6), and finally human body pictogram, which was selected by 3.9% (n=3).

**Figure 5:**
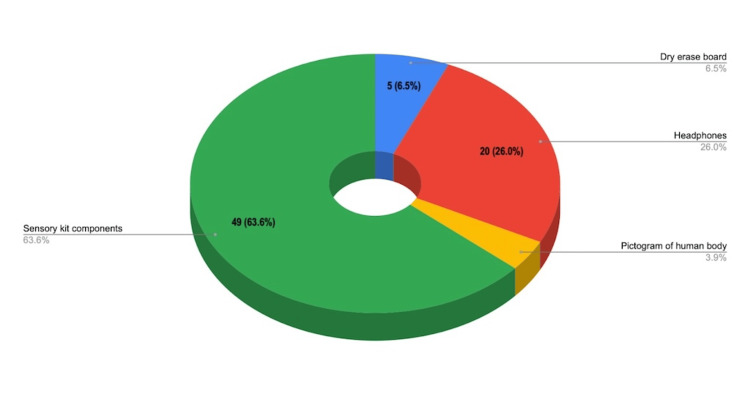
Most Useful Reported Component of BBB Kits BBB: Ben's Blue Bags

These findings highlight the critical role of sensory tools in reducing anxiety and facilitating smoother EMS interactions with patients with ASD (see Figure 5).

Frequency of use 

**Figure 6:**
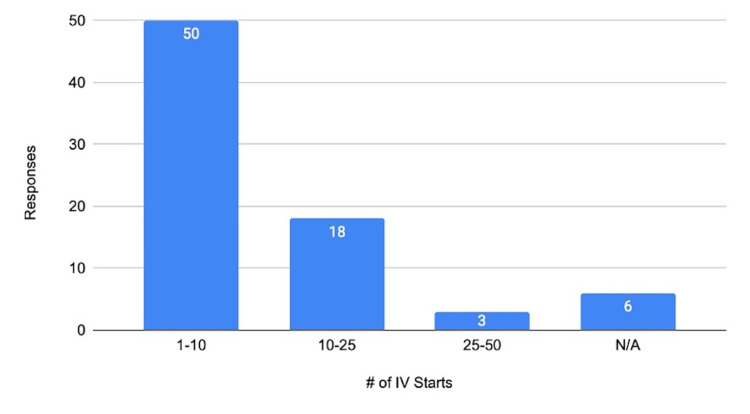
Frequency of Help Starting Intravenous (IV) Placements

Among those who found the kits helpful for starting IVs, 29.58% (n=21) reported using them in at least 10 patient encounters. The frequency of kit use varied by transport duration and location, with rural areas (longer transport times) utilizing the kits more frequently (see Figure 6).

## Discussion

ASD is a neurodevelopmental condition that affects social interactions, communication, learning, and development. Individuals with ASD often exhibit irritability, struggle with even slight deviations from their routine, and demonstrate hypersensitivity to sensory inputs such as light, sound, texture, or temperature [7]. The nature of EMS - with its sirens, flashing lights, and various medical equipment - can exacerbate stress in this population, leading to challenging interactions during emergency situations [5,6].

In the United States, approximately 2.2% of eight-year-olds and 2.3% of adults meet Diagnostic and Statistical Manual of Mental Disorders (DSM) criteria for ASD, making encounters with EMS personnel relatively common [7]. While research on ASD in prehospital settings remains limited, studies have shown that adolescents with ASD utilize emergency departments (ED) four times more frequently than their non-ASD peers. Furthermore, these individuals have a 3.7 times higher rate of hospital admission during ED visits [8]. Data suggests that families of patients with ASD find the provision of general healthcare challenging, dissatisfying, and burdensome both emotionally and financially [9]. This study demonstrates the effectiveness of the “Ben’s Blue Bags” (BBB) program in addressing these challenges. The overwhelmingly positive feedback from EMS providers, with a mean effectiveness rating of 9.08, underscores the value of sensory aids and communication tools in improving care for patients with ASD. 

The study found that 94.2% of EMS personnel reported an improvement in their ability to deliver high-quality care to individuals with ASD when using BBBs. Notably, the program contributed to the avoidance of unnecessary emergency room visits in 77.9% of the reported cases. These findings are consistent with previous research, which highlights the importance of environmental adaptations in healthcare settings for individuals with ASD [8]. For instance, studies have shown that sensory-friendly modifications, such as those provided in the BBB kits, can reduce anxiety and improve cooperation during medical interventions ​[9].

The BBB program aligns with the broader movement toward neurodiversity in healthcare, emphasizing the need for individualized and patient-centered care. By equipping EMS providers with tools tailored to the unique needs of patients with ASD, the program promotes inclusivity and enhances the quality of prehospital care. This approach is in line with the principles of universal design in healthcare, which seek to accommodate diverse patient needs without requiring specialized knowledge from every provider.

Limitations

Although the results are promising, this study has several limitations. First, the survey was conducted within a single EMS system, limiting the generalizability of the findings. The geographic focus on Northwest Indiana may not reflect the experiences of EMS providers in other regions with different patient demographics or healthcare systems. Second, the self-reported nature of the survey data introduces potential biases, such as social desirability bias or recall bias. Additionally, the study did not include a control group to compare outcomes with standard care practices. 

Future research

Future research should aim to replicate these findings in diverse settings to evaluate the broader applicability of sensory aids in EMS care. Comparative studies with control groups could provide more robust evidence of the impact of BBBs on patient outcomes. Moreover, longitudinal studies could explore the long-term benefits of sensory aids, including their role in reducing healthcare costs by minimizing unnecessary ER visits and hospitalizations.

## Conclusions

ASD is a prevalent condition in the United States that presents unique challenges for EMS crews in prehospital settings. Innovative approaches are needed to improve care for these patients and reduce the significant personal and societal costs associated with these encounters. Sensory bags, like BBBs, represent one such approach, though they are not yet widely used or studied. This study demonstrates the potential utility of these tools in improving outcomes for individuals with ASD. It strongly advocates for their expanded use and emphasizes the need for further research on their impact within both EMS and emergency department settings.
